# High Breakdown Voltage and Low Dynamic ON-Resistance AlGaN/GaN HEMT with Fluorine Ion Implantation in SiN_x_ Passivation Layer

**DOI:** 10.1186/s11671-019-3025-8

**Published:** 2019-06-04

**Authors:** Chao Yang, Xiaorong Luo, Tao Sun, Anbang Zhang, Dongfa Ouyang, Siyu Deng, Jie Wei, Bo Zhang

**Affiliations:** School of Electronic Science and Engineering, State Key Laboratory of Electronic Thin Films and Integrated Devices, University of Electronic Science and Technology of China, Chengdu, 610054 China

**Keywords:** AlGaN/GaN HEMT, Fluorine ion implantation, SiN_x_ passivation layer, Breakdown voltage, Dynamic ON-resistance

## Abstract

In this study, we proposed and experimentally demonstrated a high breakdown voltage (BV) and low dynamic ON-resistance (*R*_ON, D_) AlGaN/GaN high electron mobility transistor (HEMT) by implanting fluorine ions in the thick SiN_x_ passivation layer between the gate and drain electrodes. Instead of the fluorine ion implantation in the thin AlGaN barrier layer, the peak position and vacancy distributions are far from the two-dimensional electron gas (2DEG) channel in the case of fluorine ion implantation in the thick passivation layer, which effectively suppresses the direct current (DC) static and pulsed dynamic characteristic degradation. The fluorine ions in the passivation layer also extend the depletion region and increase the average electric field (E-field) strength between the gate and drain, leading to an enhanced BV. The BV of the proposed HEMT increases to 803 V from 680 V of the conventional AlGaN/GaN HEMT (Conv. HEMT) with the same dimensional parameters. The measured *R*_ON, D_ of the proposed HEMT is only increased by 23% at a high drain quiescent bias of 100 V, while the *R*_ON, D_ of the HEMT with fluorine ion implantation in the thin AlGaN barrier layer is increased by 98%.

## Background

In recent decades, novel semiconductor materials, such as GaN, metal oxides, and 2D materials, have been widely studied to further enhance the energy conversion and storage efficiency, owing to their superior material and device properties [1–8]. Among them, GaN-based AlGaN/GaN high electron mobility transistors (HEMTs) are good candidates for high power, high frequency, and low loss applications because of high critical breakdown field and high electron mobility [9–14]. The breakdown voltage (BV) is one of the most important design targets, and the reported values are still far below the theoretical limit [15, 16]. Therefore, it is of great importance to further improve the BV, especially not at the cost of increasing the device size. Several termination techniques have been proposed to improve the BV, such as field plate [17–19], fluorine ion implantation [20–22], and recessed gate-edge termination [23, 24]. Fluorine ions implanted in the thin AlGaN barrier layer (FBL) [22] has a simple fabrication process without inducing an additional parasitic capacitance; however, the peak position of the fluorine profile and vacancy distributions is near to the two-dimensional electron gas (2DEG) channel, which would inevitably cause significant static and dynamic characteristic degradation.

In this work, a high breakdown voltage and low dynamic ON-resistance (*R*_ON, D_) AlGaN/GaN HEMT with fluorine ion implantation in the SiN_x_ passivation layer (FPL HEMT) is experimentally investigated. Unlike in the case of the fluorine ion implantation in the thin AlGaN barrier layer, fluorine ion implantation in the thick passivation layer could keep the peak position of fluorine and vacancy distributions far away from the 2DEG channel, thus effectively suppress the static and dynamic characteristic degradation. Fluorine ions in the passivation layer as a termination technique are also used to optimize the surface electric field (E-field) distribution, thus achieving an enhanced BV. In conclusion, the FPL HEMT demonstrates excellent static characteristics and dynamic characteristics.

## Fabrication Methods

**Fig. 1:**
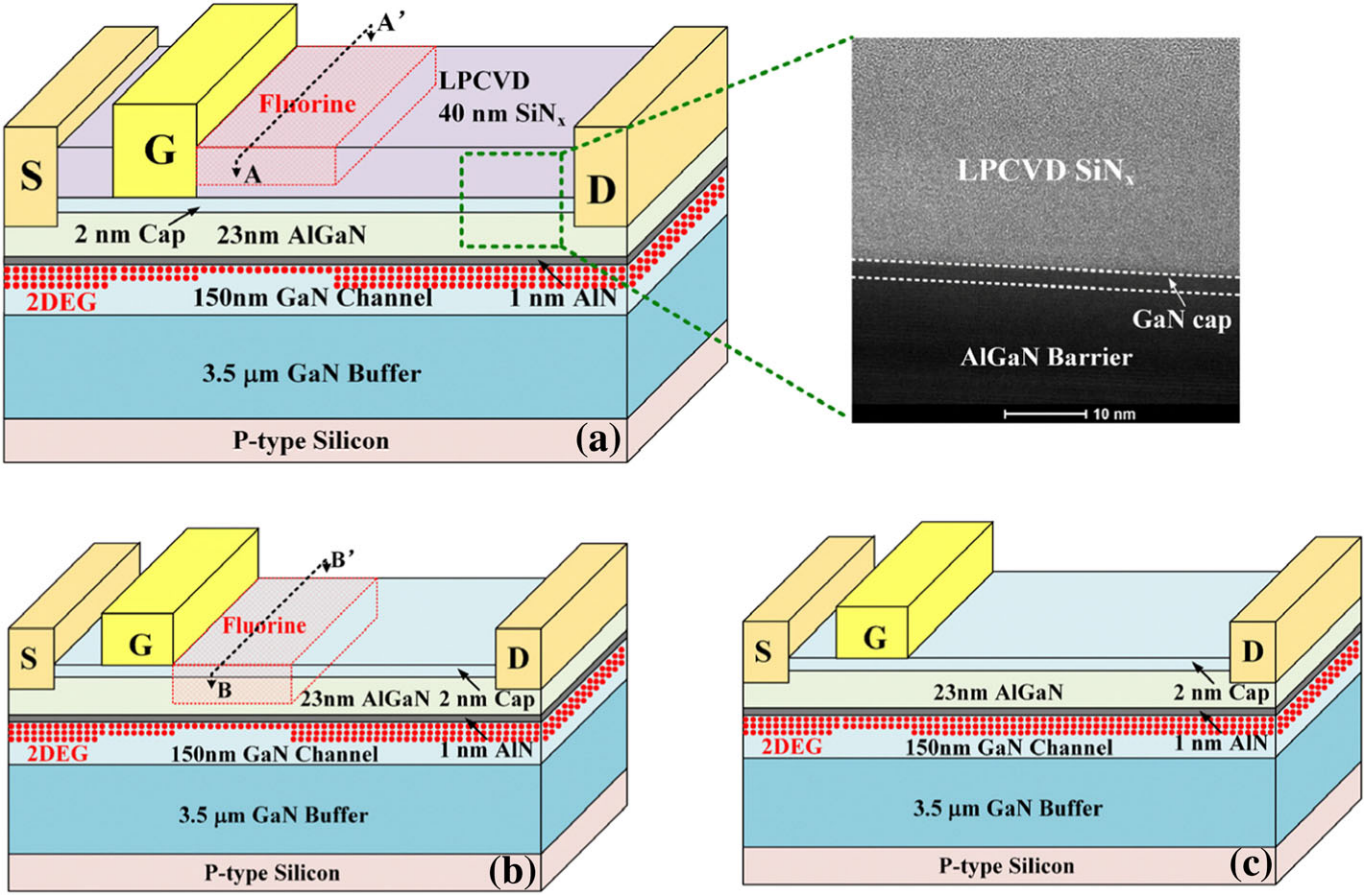
Three-dimensional schematic of **a** FPL HEMT (inset: HR-TEM micrograph of LPCVD SiN_x_), **b** FBL HEMT, and **c** Conv. HEMT

Figure 1 is the three-dimensional schematic of FPL HEMT, FBL HEMT, and Conv. HEMT, respectively. All devices feature a gate length *L*_G_ of 2.5 μm, a gate-source distance *L*_GS_ of 1.5 μm, and a gate-drain distance *L*_GD_ of 10 μm. The epitaxial AlGaN/GaN heterostructure used for fabricating the FPL HEMT was grown on 6-in (111) silicon substrate by metal organic chemical vapor deposition (MOCVD). The epitaxial layers consist of a 2-nm GaN cap, 23-nm Al_0.25_Ga_0.75_N barrier, 1-nm AlN interlayer, 150-nm GaN channel, and 3.5-μm GaN buffer. The Hall effect measured density and mobility of the 2DEG were 9.5 × 10^12^ cm^−2^ and 1500 cm^2^/V s, respectively. The proposed FPL HEMT started with mesa isolation which was implemented by a high power Cl_2_/BCl_3_-based inductively coupled plasma (ICP) etching. Then, a 40-nm-thick low pressure chemical vapor deposition (LPCVD) SiN_x_ layer was deposited at 780 °C/300 mTorr with a NH_3_ flow of 280 sccm and a SiH_2_Cl_2_ flow of 70 sccm, yielding a deposition rate of 3.7 nm/min. The refractive index is measured by ellipsometer as 1.978, and the N/Si ratio of SiN_x_ is 1.31 [25]. The crystallinity of LPCVD SiN_x_ is amorphous, which is confirmed by high-resolution transmission electron microscope (HR-TEM) micrograph (see the inset of Fig. 1a). After opening the source and drain contact windows by SF_6_ plasma dry etching, the Ti/Al/Ni/Au (20/150/45/55 nm) ohmic contact was deposited and annealed at 890 °C for 30 s in N_2_ ambient. The contact resistance of 1 Ω mm and sheet resistance of 400 Ω/square were extracted by the linear transmission line method. Next, the gate metal electrode is formed by Ni/Au (50 nm/150 nm) deposition and lift-off process. Then, the fluorine ion implantation window (Length of window = 3 μm) is formed by AZ5214 photoresist, and fluorine ions were implanted by SEN NV-GSD-HE ion implanter at an energy of 10 keV at a dose of 1 × 10^12^ cm^−2^. Finally, the samples were annealed at 400 °C for 15 min in N_2_ ambient to complete the transistor fabrication flow [26].

## Results and Discussion

**Fig. 2:**
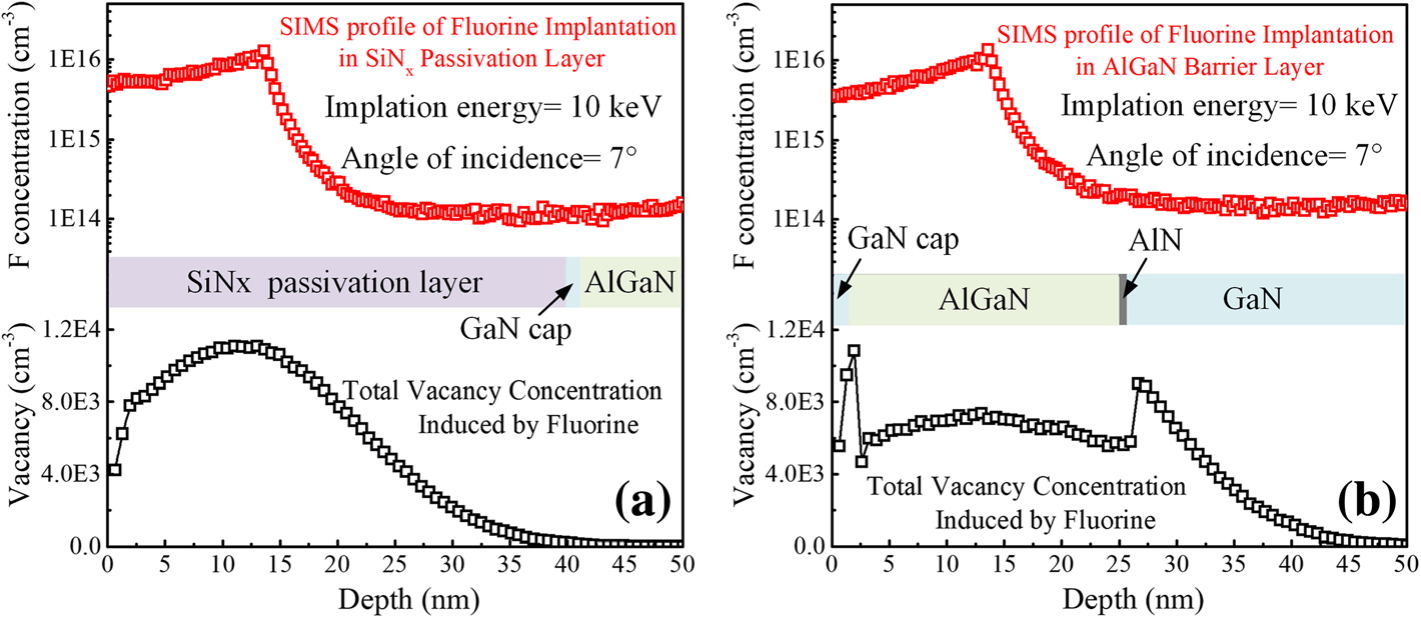
Measured SIMS profile of fluorine ion concentration and simulated vacancy concentration by TRIM along the cut lines. **a** *A-A′.* **b** *B-B′*

Figure 2 shows the measured secondary ion mass spectroscopy (SIMS) profile of fluorine ion concentration and simulated vacancy concentration by TRIM along the cut lines: (a) *A-A′* of FPL HEMT and (b) *B-B′* of FBL HEMT, respectively. At the same energy and dose of fluorine ion implantation, the measured peak position from the surface and maximum concentration of the fluorine profile is almost the same for the two structures. In the case of the fluorine ion implantation in the thin AlGaN barrier layer, the vacancies induced by fluorine extend to the 2DEG channel region. The distribution of vacancy concentration is discontinuous at each interface because the bond energy of every material is different [27]. However, in the case of the fluorine ion implantation in the thick SiN_x_ passivation layer, the vacancy distribution is located within the SiN_x_ passivation layer and far from the 2DEG channel. The vacancies induced by ion implantation would trap the 2DEG channel, and 2DEG could be easily trapped if the vacancy distribution is near to the 2DEG [28]. In conclusion, fluorine ion implantation in the thick SiN_x_ passivation layer could significantly reduce the influence of ion implantation on the 2DEG channel and suppress the static and dynamic characteristic degradation effectively.

**Fig. 3:**
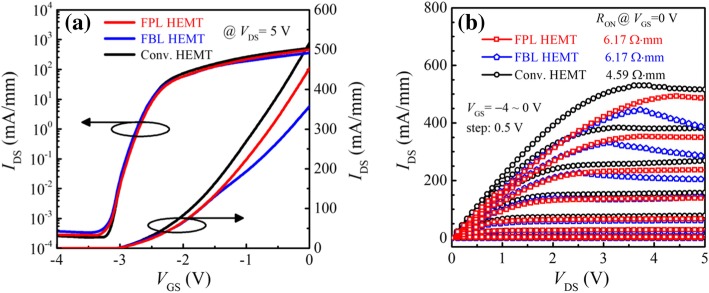
Measured **a** *I-V* transfer characteristics, and **b** DC output characteristics for three HEMTs

Figure 3 illuminates the measured *I-V* transfer characteristics and direct current (DC) output characteristics. Compared with the Conv. HEMT, both the FPL HEMT and FBL HEMT show a decrease in *I*_DS_ and an increase in static ON-resistance (*R*_ON_), because the fluorine ions cause the assisted depletion of the 2DEG in the drift region and thus decrease the 2DEG density [29]. In addition, the ion implantation also decreases the 2DEG mobility. The Hall effect measured 2DEG mobilities of the FPL and FBL HEMTs are 228 cm^2^/V s and 203 cm^2^/V s after ion implantation, respectively. Owing to the same dose of fluorine ions, the output characteristics and *R*_ON_ of FPL HEMT and FBL HEMT are almost the same at a low drain voltage (e.g., *V*_DS_ < 3 V). However, when *V*_DS_ > 3 V, the saturation drain current collapse occurs in the FBL HEMT, because the vacancy profile of fluorine extends to the 2DEG channel region, and the 2DEG could be easily trapped by these deep level vacancies induced by fluorine when drain voltage is large than critical drain voltage (e.g., *V*_DS_ > 3 V) [30], thereby decreasing the drain current. The vacancy distribution of FPL HEMT is far from the 2DEG channel, thus suppressing the saturation drain current collapse effectively.

**Fig. 4:**
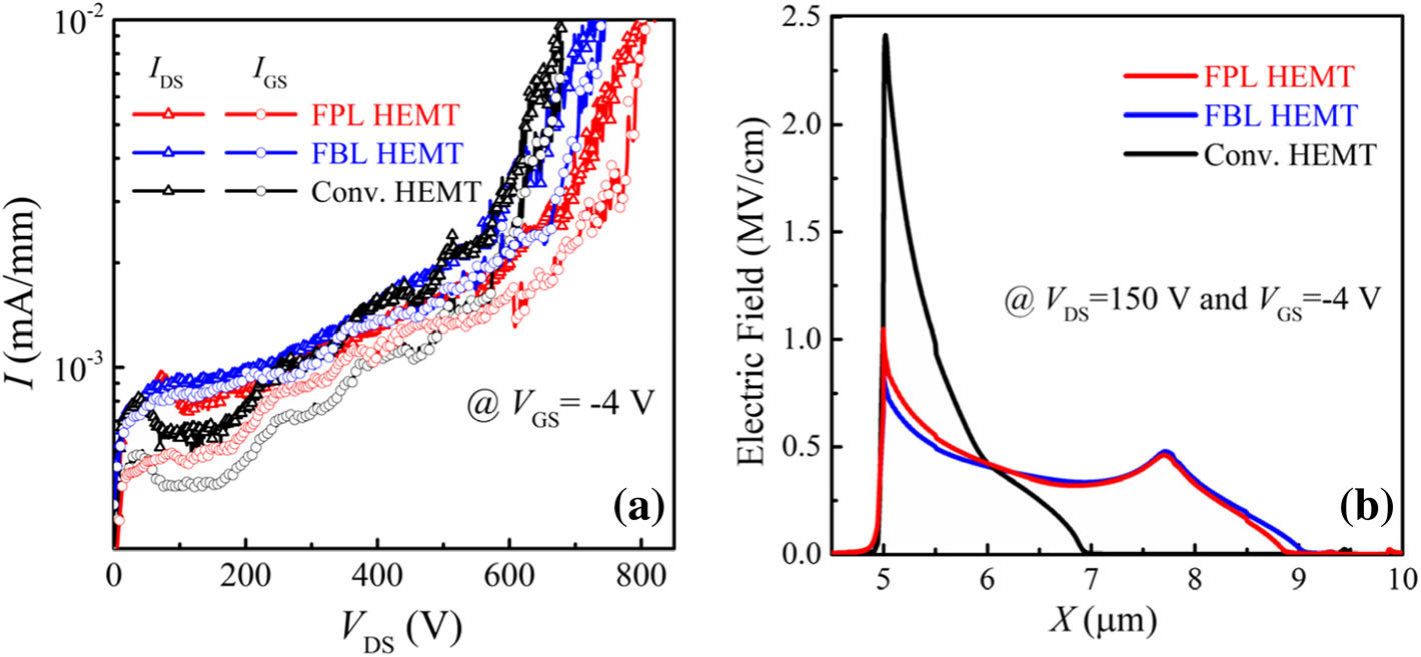
**a** Measured off-state *I-V* characteristics with a gate voltage of−4 V, keeping the substrate floated. **b** Simulated surface E-field distributions at *V*_DS_ = 150 V

Figure 4 shows the measured *I-V* characteristics and simulated surface E-field distributions on the blocking state. The BVs of the FPL/FBL/Conv. HEMTs are 803/746/680 V, respectively. The BV is defined as the drain-source voltage at the drain current (*I*_DS_) of 1 μA/mm with *V*_GS_ = − 4 V. The fluorine ions between the gate and drain as a termination technique reduce the E-field peak at the gate edge and cause a new E-field peak at the end of ion implantation region, and thus, FPL HEMT and FBL HEMT achieve more uniform surface E-field distribution and higher BV than that of the Conv. HEMT. Compared with FPL HEMT, FBL HEMT has an enhanced electric field modulation effect, because the fluorine ion profile is near to the 2DEG channel. However, for the FBL HEMT, ion implantation would inevitably induce additional damages in AlGaN barrier [31, 32], leading to a continuous gate leakage current path of *gate-barrier layer-2DEG*; therefore, the BV of FBL HMET is slightly smaller than that of the FPL HEMT.

**Fig. 5:**
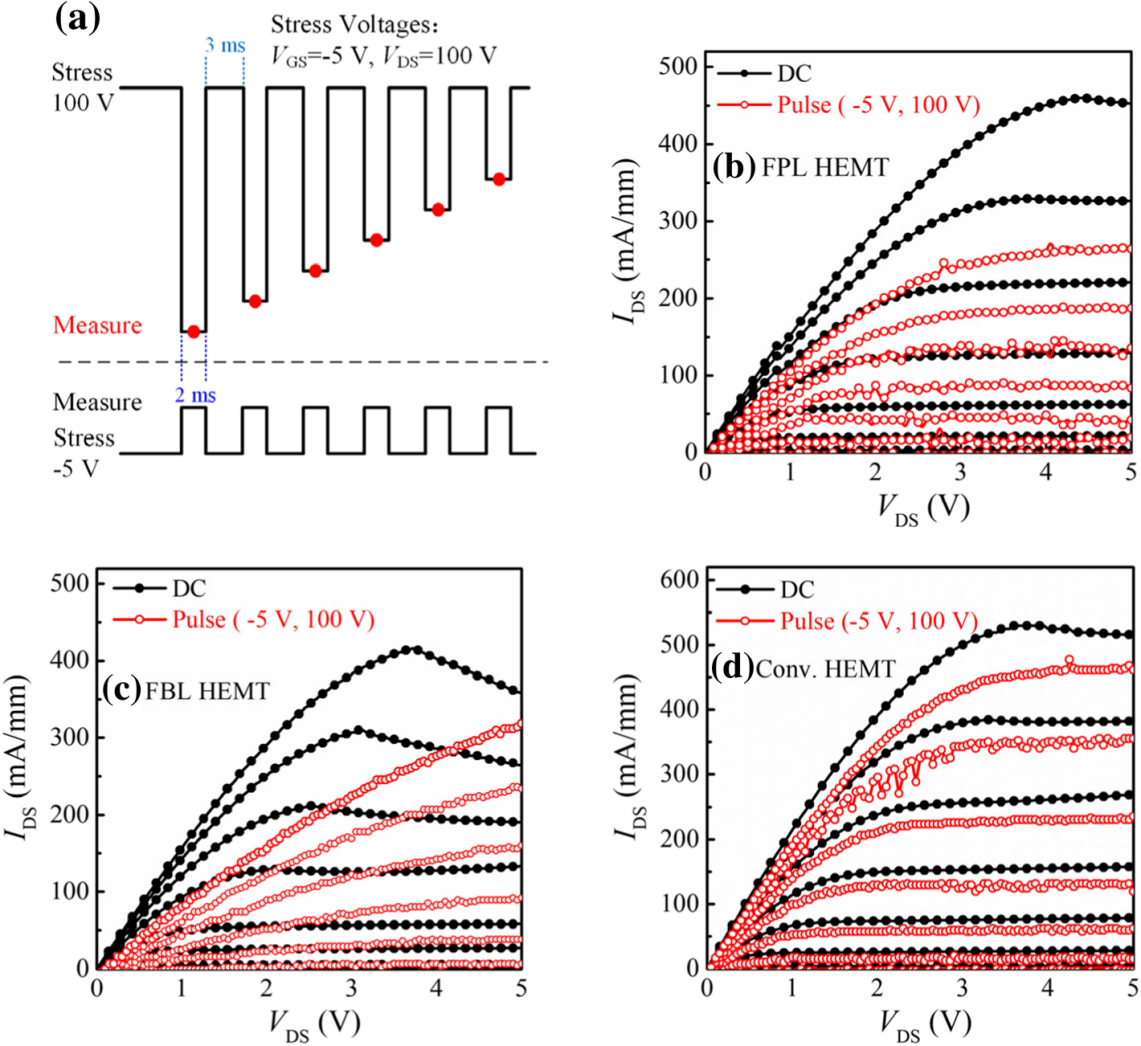
**a** Schematic depicting application of stress voltage during the pulsed *I*_DS_-*V*_DS_ measurements. Pulsed *I*_DS_-*V*_DS_ characteristics of the fabricated AlGaN/GaN HEMTs with **b** FPL HEMT, **c** FBL HEMT, and **d** Conv. HEMT (*V*_GS_ = − 4~0 V; step: 0.5 V)

**Fig. 6:**
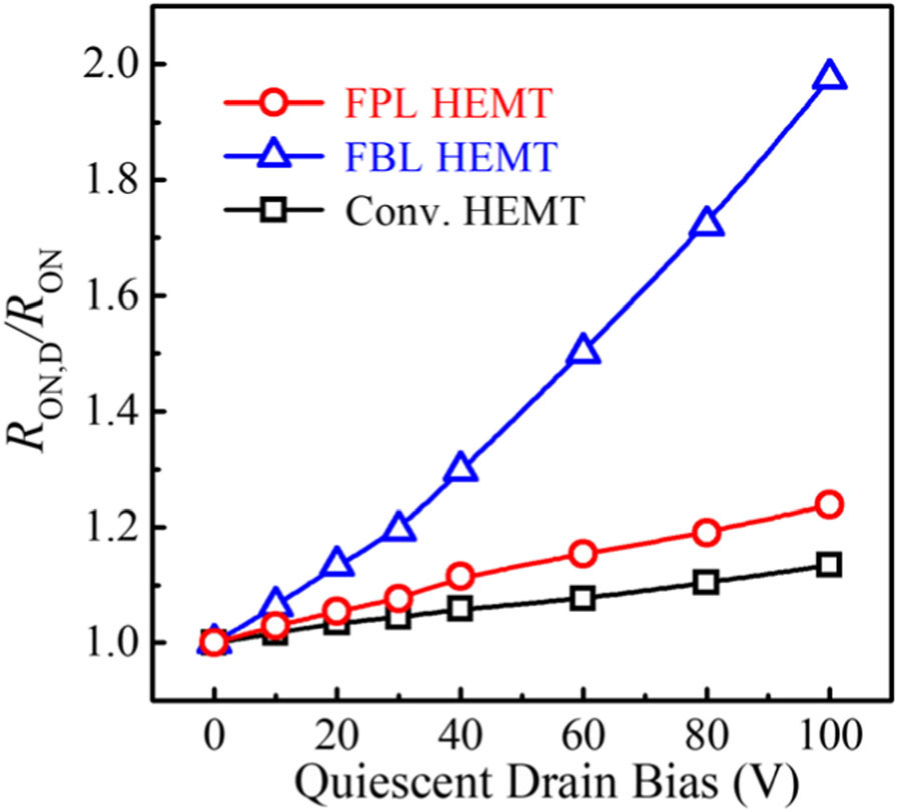
Ratio of *R*_ON, D_/*R*_ON_ for the fabricated HEMTs at different quiescent drain bias points. The pulse width and period are 3 ms and 5 ms, respectively

Pulsed *I*_DS_-*V*_DS_ measurements [33] under slow switching were performed to characterize the behavior of dynamic ON-resistance (*R*_ON, D_) of the fabricated AlGaN/GaN HEMTs. Figure 5a is the schematic depicting the application of stress voltage during the pulsed *I*_DS_-*V*_DS_ measurements. In pulsed *I-V* measurements, the gate and drain electrodes of the GaN HEMTs were subjected to short voltage pulses before each *I-V* measurement to ensure that the devices were in the off-state. The pulse width is 3 ms and the period is 5 ms. Figure 5 b–d show the comparison of the pulsed output characteristics of the devices under (*V*_GS0_, *V*_DS0_) of (0 V, 0 V) and (0 V, 100 V). It can be seen that there is a slightest degradation (12.3%) of dynamic ON-resistance for the Conv. HEMT, owing to the absence of fluorine ion implantation process. In comparison with FBL HEMT, FPL HEMT has a low degradation of dynamic ON-resistance. Owing to the passivation layer, the vacancy distribution is far away from the 2DEG channel and is located within the passivation layer, which suppresses the charge trapping in the FPL HEMT. Figure 6 summarizes the ratio values of *R*_ON, D_/*R*_ON_ for the three HEMTs under (*V*_GS0_, *V*_DS0_) from (0 V, 0 V) and (0 V, 100 V) at a step of 20 V. For the FBL HEMT, the measured *R*_ON, D_ is already increased by 98% of the static one at (*V*_GS0_, *V*_DS0_) of (0 V, 0 V) and (0 V, 100 V), while the *R*_ON, D_ of the FPL HEMT is increased by only 23% at a high drain quiescent bias of 100 V.

## Conclusions

In conclusion, we proposed a novel AlGaN/GaN HEMT with a high breakdown voltage and low dynamic ON-resistance. It features fluorine ion implantation in the thick SiN_x_ passivation layer. Fluorine ion implantation in passivation layer could effectively suppress electrical characteristic degradation. The dynamic ON-resistance is only 1.23 times as larger as the static ON-resistance after off-state *V*_DS_ stress of 100 V, while it is 1.98 times for the FBL HEMT. In addition, the fluorine ions in the passivation layer also modulate the E-filed distribution and spread the depletion region; thus, the BV of the proposed HEMT increases to 803 V from 680 V of conventional AlGaN/GaN HEMT.

## Data Availability

All data generated or analyzed during this study are included in this published article.

## Abbreviations

2DEG: Two-dimensional electron gas
HEMT: High electron mobility transistor
ICP: Inductively coupled plasma
LPCVD: Low pressure chemical vapor deposition
MOCVD: Metal organic chemical vapor deposition
SIMS: Secondary ion mass spectroscopy
TEM: Transmission electron microscope
